# Supplementary material for: Detection of pulmonary nodules based on a multiscale feature 3D U-Net convolutional neural network of transfer learning
Source: PLoS One. 2020 Aug 26;15(8):e0235672. doi: 10.1371/journal.pone.0235672 (PMC7449493; doi:10.1371/journal.pone.0235672)
Supplement: S1 Dataset — (DOCX) [file pone.0235672.s001.docx]

All DICOM files are available from the LUNA16 and TIANCHI17 database.

http://academictorrents.com/collection/luna-lung-nodule-analysis-16---isbi-2016-challenge

Tianchi medical AI competition: intelligent diagnosis of pulmonary nodules[DB/OL].

https://tianchi.aliyun.com/competition/entrance/231601/information

The data are presented as follows：

The contest dataset provided thousands of low-dose lung CT images (MHD format) of high-risk patients,each image contains a series of multiple axial sections of the thorax. The number of slices contained in each image will vary, The original image is 3D image, the 3D image is composed of a large number of 2D images of multiple axial slices. The number of 2D images can vary based on different factors, such as scanning machines and patients. After registering as a member, users can download the preliminary contest database, The specific data are as follows:

1. Data volume: 1000 patients, all with nodules.
2. Data quality:
3. The size of the nodules are roughly distributed as follows:

| 10-30mm | 5-10mm |
| --- | --- |
| 50% | 50% |

1. The pathological analysis nodules were not included, all other nodules were identified by three doctors.
2. The data format:

CT image: MHD format.

1. Nodule labeling information:

CSV file, marking the location and size of the nodules(mm).

| seriesuid | coordX | coordY | coordZ | diameter_mm |
| --- | --- | --- | --- | --- |
| LKDS_00001 | -100.56 | 67.26 | -231.81 | 6.44 |

1. Seam thickness (mm)

The thickness of all CT images is less than 2mm.
