# Supplementary material for: Detection of pulmonary nodules based on a multiscale feature 3D U-Net convolutional neural network of transfer learning
Source: PLoS One. 2020 Aug 26;15(8):e0235672. doi: 10.1371/journal.pone.0235672 (PMC7449493; doi:10.1371/journal.pone.0235672)
Supplement: Attachment — Submitted filename: PONE-D-19-32789_reviewer.doc [file pone.0235672.s002.doc]

**General Comments**

***General:***

***In this paper, the authors reported a new computer-aided detection scheme is proposed, the 3D U-Net convolutional neural network to automatically detect pulmonary nodules from the thoracic region containing background and noise. In this work, the method solved not only the problem that a small dataset was difficult to train a deep model but also the overfitting problem. Moreover, layer by layer transfer training method improved the accuracy of image detection under the condition of small samples. The topic of the manuscript is interested and useful for audience. The reviewer only has some suggestions as follows:***

**Specific comments**

1. ***Why only choose to rotate the image at 90°,180°,270°degrees in data enhancement?***
2. ***Can you get better results if you use shifting, shearing other methods in the data enhancement part?***
3. ***In Fig. 7, how to determine the parameters of 3D U-Net model have been trained properly, and can be transfer to another model?***
4. ***Why choose 3D U-NET instead of other 3D CNN models?***
5. ***In page 14, the explanation of cascading layer could better repair the details of the target should be more clarified.***
6. ***Please rearrange the order of the figure (ex. Fig 9(c) 9(d)?).***
