# Supplementary material for: Detection of pulmonary nodules based on a multiscale feature 3D U-Net convolutional neural network of transfer learning
Source: PLoS One. 2020 Aug 26;15(8):e0235672. doi: 10.1371/journal.pone.0235672 (PMC7449493; doi:10.1371/journal.pone.0235672)
Supplement: Attachment — Submitted filename: Response to Reviewers.doc [file pone.0235672.s003.doc]

**General Comments**

***General:***

***In this paper, the authors reported a new computer-aided detection scheme is proposed, the 3D U-Net convolutional neural network to automatically detect pulmonary nodules from the thoracic region containing background and noise. In this work, the method solved not only the problem that a small dataset was difficult to train a deep model but also the overfitting problem. Moreover, layer by layer transfer training method improved the accuracy of image detection under the condition of small samples. The topic of the manuscript is interested and useful for audience. The reviewer only has some suggestions as follows:***

**Specific comments**

1. ***Why only choose to rotate the image at 90°,180°,270°degrees in data enhancement?***

***The rotation Angle of the specification is easy to achieve algorithmically, and it also achieves the purpose of obtaining more images from different angles.***

2. ***Can you get better results if you use shifting, shearing other methods in the data enhancement part?***

***A shift can increase the amount of data, Since most pulmonary nodules are small in size, incomplete pulmonary nodules are easily obtained by shearing.***

3. ***In Fig. 7, how to determine the parameters of 3D U-Net model have been trained properly, and can be transfer to another model?***

***The stochastic gradient descent optimization method (SGD) is used to fine-tune the weight, the network model can obtain more features of pulmonary nodules. the adjusted network model can be transfer to the new data set to detect pulmonary nodules.***

4. ***Why choose 3D U-NET instead of other 3D CNN models?***

***The improved multi-scale 3D U-NET network model can solve the problem of missing the low-level characteristics of pulmonary nodules in the process of network transmission, High-level features are acquired by convolution and pooling operations,high speed flow of feature information between input and output layers is achieved by using dense network and multi-scale features are generated combining with expanded convolution to improve the utilization of low-level features of pulmonary nodules,The detection accuracy of small nodules are also improved, so this paper used 3D U-NET model to detect pulmonary nodules.***

5. ***In page 14, the explanation of cascading layer could better repair the details of the target should be more clarified.***

***The cascade layer merges the output layer of the first layer with the input layer of the second, It's easy to lose the boundary pixel information in the convolution operation, The cascade layer can obtain more feature information of the target area through the combination of the high-level feature and the low-level feature, which can achieve the purpose of better repairing the target details.***

6. ***Please rearrange the order of the figure (ex. Fig 9(c) 9(d)?).***

***The figure has been rearranged.***
